# Malicious Network Behavior Detection Using Fusion of Packet Captures Files and Business Feature Data

**DOI:** 10.3390/s21175942

**Published:** 2021-09-03

**Authors:** Mingshu He, Xiaojuan Wang, Lei Jin, Bingying Dai, Kaiwenlv Kacuila, Xiaosu Xue

**Affiliations:** 1 School of Electronic Engineering, Beijing University of Posts and Telecommunications, Beijing 100876, China; hemingshu@bupt.edu.cn (M.H.); klc@bupt.edu.cn (K.K.); xxs@bupt.edu.cn (X.X.); 2 School of Computer Science, Beijing University of Posts and Telecommunications, Beijing 100876, China; jinlei@bupt.edu.cn; 3 Department of Statistics, Colorado State University, Fort Collins, CO 80523, USA; Bingying.dai@colostate.edu

**Keywords:** malicious behavior detection, data fusion, network traffic, convolution dimension

## Abstract

Information and communication technologies have essential impacts on people’s life. The real time convenience of the internet greatly facilitates the information transmission and knowledge exchange of users. However, network intruders utilize some communication holes to complete malicious attacks. Some traditional machine learning (ML) methods based on business features and deep learning (DL) methods extracting features automatically are used to identify these malicious behaviors. However, these approaches tend to use only one type of data source, which can result in the loss of some features that can not be mined in the data. In order to address this problem and to improve the precision of malicious behavior detection, this paper proposed a one-dimensional (1D) convolution-based fusion model of packet capture files and business feature data for malicious network behavior detection. Fusion models improve the malicious behavior detection results compared with single ones in some available network traffic and Internet of things (IOT) datasets. The experiments also indicate that early data fusion, feature fusion and decision fusion are all effective in the model. Moreover, this paper also discusses the adaptability of one-dimensional convolution and two-dimensional (2D) convolution to network traffic data.

## 1. Introduction

Cyber security plays an indispensable role in people’s life. Malicious network behaviors will affect account security, software security, payment security, communication security and so on in daily life [1]. However, with the continuous development of application technology, cyber security threats are becoming increasingly complex. Thus, it is more laborious to identify malicious behaviors on the Internet [2]. Some application systems and frameworks for enhancing information security have also been covered by many researchers [3,4,5]. Some researchers have tried discovering more features in each network application behavior and tried identifying the malicious ones. However, the feature extraction of a single method is always very limited. By assuming that there are different types of data samples which can represent network behaviors in different forms, we can collect dissimilar information from each one. If this information can be combined to build detection models, the identification results may achieve better performance. In this paper, fusion models of packet captures files and business feature data are used to achieve better malicious network behavior detection results.

There are two common approaches for malicious network behavior detection: traditional business methods with pattern setting and ML-based ones [6]. Traditional detection methods based on fixed service characteristics are relatively simple, which always include Deep Packet Inspection (DPI)-based, port-based and statistic-based ones [7,8,9]. However, due to its single mode, the detectable malicious behavior is relatively fixed, and they are less and less used in today’s applications. As the ML-based technology continues to mature in daily application fields such as computer vision (CV), natural language processing (NLP) and intelligent recommendation, malicious behavior detection methods based on ML and DL are used more frequently. Generally, traditional ML-based methods using business features and DL-based methods utilizing raw data collected from traffic include two major methods. As for traditional ML-based methods, which usually consider the features concluded by business experts, they lack generalization ability that makes it difficult to detect some malicious attacks with huge fluctuations. As for DL-based methods, they always extract features automatically. Therefore, some attacks with a set business pattern may not be identified. Moreover, these simple models always address one type of data source for a network behavior sample. If there are over two types of data, some effective features will not be mined while only one type of data is used.

Fusion-based models are widely used in Human Action Recognition (HAR) and achieved good performance [10,11,12]. It frequently happens that HAR uses pictures with human’s action to complete the recognition process. RGB and depth images are two common types of original data for this application. In addition to these vision data, some wearable inertial data outputs from sensors also play an important role in HAR. For each type of data source, it contains relatively unitary information in a certain aspect. Then, fusion models based on DL can solve this problem. Two or more types of data can be fused through early data fusion, feature fusion and decision fusion. Furthermore, researchers show that fusion methods are really effective in HAR. In malicious behavior detection tasks, a few approaches use fusion frameworks to complete this process. Compared with HAR data, the recording method of network access is relatively single. Generally speaking, network traffic and some business feature-based data are two common data sources [13]. This paper conducted these two kinds of data in order to accomplish fusion detection experiments and made some progress. The key contributions of the presented work are the following:

(1) We proposed a Convolutional Neural Network(CNN)-based fusion model of packet capture (PCAP) files and business feature data for malicious network behavior detection, and experiments results show that the fusion model can improve the detection precision with little increase in time and resource consumption.
(2) Three fusion approaches, early data fusion, feature fusion and decision fusion, are discussed here, and we find that each method can extract more discriminating and complementary features. On the whole, feature fusion and decision fusion have better performances in the datasets.
(3) Different from image or video data, traffic network and business feature data lack two-dimensional association information. In this paper, we compared the effect of one-dimensional and two-dimensional convolution on network malicious behavior detection scene. The results show that one-dimensional convolution structures have a better performance.
(4) We evaluated the proposed fusion methods on different datasets and compared the effects with previous studies. Experimentally, the results illustrated that our methods can adapt to various network data in lots of different scenarios.

The rest of the paper is organized as follows. Section 2 introduces some methods and applications related to this paper. Section 3 depicts the proposed methods and explains some significant concepts. In Section 4, we illustrate the experiment process and results. In addition, we compare the outputs and analyze the results accordingly. Section 5 provides the conclusions of this paper.

## 2. Related Work

Since few methods use fusion frameworks in network behavior detection, for the purposes of better explaining and understanding, we discuss some fusion models that perform well in other application fields. Furthermore, this section depicts some ML-based behavior detection approaches using PCAP files and business feature data.

Traditional ML-based methods account for a large proportion of current malicious network behavior detection applications. These methods focus on the processed business feature data and make some progress, the results of which are easier trusted by experts. Waskle et al. [14] proposed an approach to develop an efficient intrusion detection system (IDS) by using principal component analysis (PCA) and random forest (RF) algorithm, and the results performed better than the basic traditional ML models such as Naive Bayes and decision tree (DT). Lu et al. [15] combined Synthetic Minority Oversampling Technique (SMOTE) and Edited Nearest Neighbor (ENN) Rule on RF and achieved higher precision, recall and F1-score. Gao et al. [16] proposed an adaptive ensemble-learning model based on DT, RF and K-Nearest Neighbor (KNN). Marteau et al. [17] proposed an ensemble approach composed of random partitioning binary trees named DiFF-RF for malicious behavior detection. Miah et al. [18] proposed a multi-layer classification approach for imbalanced network intrusion based on cluster and RF. Table 1 lists some related literature in the past three years that contain ML-based and DL-based methods in the past three years.

Compared with traditional ML-based network network detection methods, DL-based methods rely less on business experience. DL-based models have a perfect performance on CV, NLP, automatic control(AC) and many other related application fields. In light of their research methodologies, CNN is the most popular model used here. The second half of the Table 1 introduces these methods and their contributions. Some of them show solicitude for data augmentation [22,33], and some focus on the problem of imbalanced data [22,26,27,34]. Moreover, model interpretability [23,29] and feature generation [28,32] are also the focus of attention. Although each method solves the problems in different scenarios, they have consistent goals in detection results. All of them [15,16,17,18,19,20,21,22,23,24,25,26,27,28,29,30,31,32,33,35] pay close attention to the improvement of detection accuracy and efficiency. In this paper, we also focus on the most basic and important target in intrusion detection system (IDS). The experiment results show that the proposed fusion frameworks make some significant improvements on different datasets.

It is the fusion models of other application fields that inspire us. This paragraph will introduce some related fusion models. Chen et al. [36] made a review of the publicly available datasets on HAR and compared their results. The results show that each sensor modality has its own limitations, and fusion methods can reduce the impacts. After that, some researchers invested their efforts to further verify the effectiveness of fusion methods on HAR application. Dawar and Kehtarnavaz [37] achieved the recognition of the detected actions of interest based on the fusion of depth images and inertial signals, which shows the effectiveness in dealing with realistic continuous action streams. Dawar et al. [38] used the decision fusion method of CNN for depth images and LSTM for inertial signals, and the results indicate the positive impact of decision-level fusion and the data augmentation on recognition accuracies. Through a comprehensive understanding of these methods, we attempt to concentrate on fusion methods for network detection in this paper. PCAP files and business feature data can support us in accomplishing this task.

## 3. Proposed Methods

In this section, we firstly describe the general process of malicious behavior detection based on the proposed fusion methods. Then, we depict the structures of three fusion models, namely early data fusion model, feature fusion model and decision fusion model. Finally, we introduce the comparation of one-dimensional and two-dimensional convolution structure for malicious behavior detection in our model.

### 3.1. Framework of Fusion Methods for Malicious Behavior Detection Process

**Figure 1:**
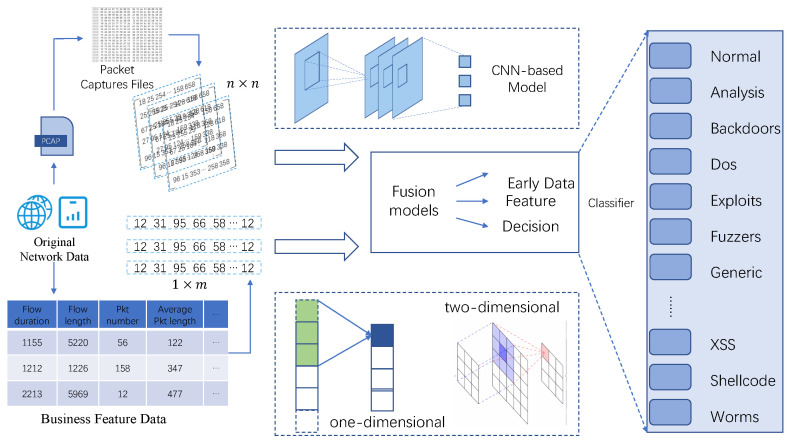
This figure depicts the process of malicious behavior detection. It contains two types of raw data collected from network traffic and three fusion methods. Detection results can be improved by using different fusion methods.

In simple terms, the proposed model aimed to collect more significant information from different types of input data. As shown in Figure 1, the input data are PCAP files and business feature data. PCAP files consist of a series of hexadecimal numbers representing different meanings, which is a little bit difficult for people to understand directly. We used CNN-based model to extract effective features automatically as the basic model before the fusion process. Business feature data comes from experts of network security field and contains a series of business feature data, which is depicted in detail in Section 4.1.2. From the method of generation, they come from machine understanding and human understanding, respectively. The availability of different source approaches make us attempt to fuse both of them and to obtain more effective attributes. Then, when data are ready, we can choose one fusion model and complete the training process. In the fusion process, we utilize one-dimensional and two-dimensional convolution structures to compare their effects on network traffic data. Finally, the classifier will identify the category based on the fusion model results.

### 3.2. Fusion Models

#### 3.2.1. Early Data Fusion Model

**Figure 2:**
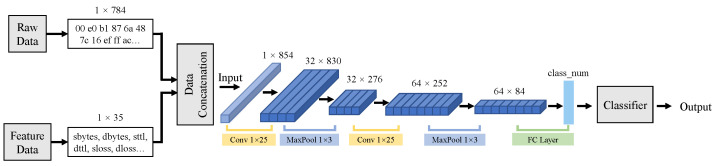
The structure of early data fusion model. The fusion process occurs in the early stages of the detection process, which can be regarded as the direct fusion of raw data information.

At first, we introduce the directest fusion model, namely early data fusion. Figure 2 depicts the structure of an early fusion model. Why call it “early” fusion process? It happens at the earliest stage of the whole process. After data processing, the PCAP and feature data can fit together as the input data of detection module. We define the PCAP raw data as $X_{1}\in R^{1\times W_{1}}$ and feature data as $X_{2}\in R^{1\times W_{2}}$, where *W* denotes the width of vectors. When early data fusion is completed, we can obtain the input data X.

(1) $$X=[X_{1},X_{2}].$$

As Equation (1) shows, data fusion is a simple and direct process for expanding information of input data. It can be considered as a data augmentation approach for each type of raw data. For model training, there are more contents that can be mined. Experimentally, we design two convolutional layers in the detection model in Figure 2. The current simple CNN-based module can achieve good performance on our datasets; thus, we did not add other more complex structures. Additionally, it is more persuasive to verify the effects of proposed malicious behavior detection fusion methods. Then, the output of the data fusion model Y can be defined as follows:

(2) $$Y=F_{classify}\left(F_{pooling\times 1\times 3}\left(F_{conv\times 1\times 25}\left(F_{pooling\times 1\times 3}\left(F_{conv\times 1\times 25}\left(X\right)\right)\right)\right)\right),$$

where *F* denotes some manipulation functions; $F_{conv\times 1\times 25}$ refers to the convolutional operation with a 25 size one-dimensional convolution kernel; $F_{pooling\times 1\times 3}$ refers to a pooling operation with 3 size pooling kernel; and $F_{classify}$ conveys a fully connected (FC) operation and an activation function, and then it outputs the classification results. Early data fusion focuses on the basic data augmentation through data connection. It helps the detection model in increasing the amount of input information at the beginning.

#### 3.2.2. Feature Fusion Model

**Figure 3:**
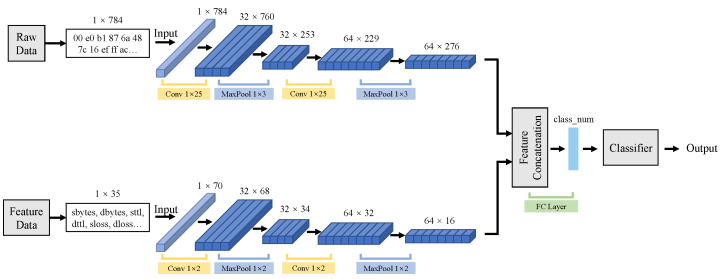
The structure of feature fusion. The fusion process occurs after the feature generation of detection model.

Feature fusion is a deep feature map fusion process. Unlike early data fusion, it is necessary to train two classifiers that have different input data in order to obtain deep feature maps. The structure is described in Figure 3. Similarly, $X_{1}$ and $X_{2}$ also denote two kinds of input data. Then, both of model $M_{1}$ and $M_{2}$ are trained, respectively. When models are ready, we define the deep future maps $N_{1}\in R^{H_{1}\times W_{1}}$ and $N_{1}\in R^{H_{2}\times W_{2}}$ from $M_{1}$ and $M_{2}$ as follows.

(3) $$N_{1}=F_{pooling\times 1\times 3}\left(F_{conv\times 1\times 25}\left(F_{pooling\times 1\times 3}\left(F_{conv\times 1\times 25}\left(X_{1}\right)\right)\right)\right),$$

(4) $$N_{2}=F_{pooling\times 1\times 2}\left(F_{conv\times 1\times 2}\left(F_{pooling\times 1\times 2}\left(F_{conv\times 1\times 2}\left(X_{2}\right)\right)\right)\right).$$

Then, the fusion deep feature matrix can be depicted as $N_{f}\mathrm{usion}$.

(5) $$N_{\mathrm{fusion}}=\left[N_{1},N_{2}\right].$$

As Equations (3)–(5) depict, feature fusion is completed before the classify layer. $N_{\mathrm{fusion}}$ will cross a fully connected layer, and the FC layer will output the final classified results Y.

(6) $$Y=F_{classify}\left(N_{\mathrm{fusion}}\right).$$

Feature fusion occurs at the model training process. It acquires trained information output from two models. It is fused after the completion of their own feature construction of two types of data. Compared with early data fusion, the interference of the two kinds of training data is lesser in the feature fusion process. When the fusion process happens, the model obtains not only the two kinds of data information but also identification abilities of two models.

#### 3.2.3. Decision Fusion Model

**Figure 4:**
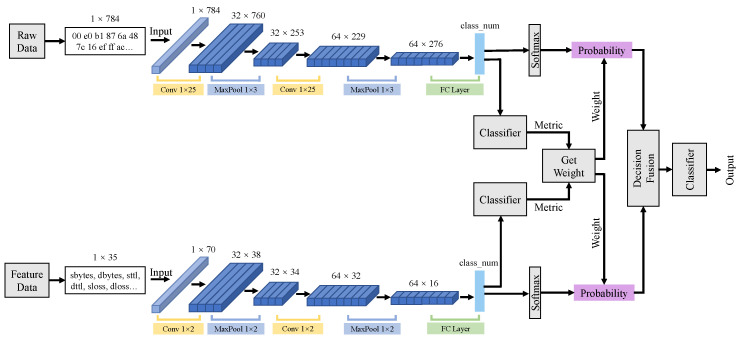
The structure of decision fusion. The fusion process occurs at the end of detection procedure.

Decision fusion occurs at the end of the classification process. Similarly to the feature fusion process, decision fusion contains two training models for PCAP data and business feature data. Figure 4 explains the structure of the decision fusion model. It is built to provide two models with different weights in the decision-making process. Considering that two kinds of input data may have different impacts on the final detection results through the training of two models, we introduced the weight $S=[S_{1}^{1},S_{1}^{2},...S_{i}^{j}]$ of model detection on each category for decision fusion. i∈{1,2} refers to two submodels, and j∈{1,2,...,j} refers to different categories in detection targets. $S_{i}^{j}$ outputs from the last FC layers of two models and represents the detection probability of category *j* of model *i*. As Figure 4 shows, decision fusion selects the highest decision score $D_{j}$ as the final precision that is calculated by weight addition. The decision score matrix D can be calculated as follows:

(7) $$D=P_{1}\times S_{1}+P_{2}\times S_{2},$$

where $P_{1}$ and $P_{2}$ denote accuracy of $M_{1}$ and $M_{2}$ in training process. They represent the detection performance of each model.

The final detection results Y=D. We can obtain the final detection output *Y*.

(8) Y=Max(D).

Unlike early data fusion and feature fusion, decision fusion artificially deals with the different influence of the two single models’ detection ability instead of automatic model mining.

### 3.3. Structures of One-Dimensional Convolution and Two-Dimensional Convolution

**Figure 5:**
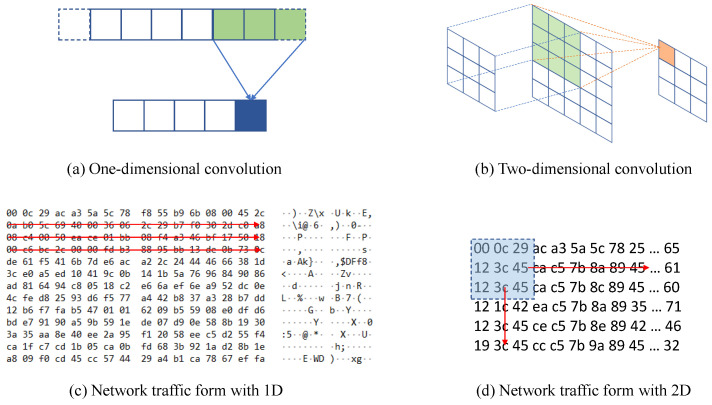
The structure of 1D and 2D convolution on network traffic data.

Due to the popularity of computer vision, two-dimensional convolution has the widest application. It is also widely used in other related fields because of its great achievements. In this work, we attempted to use 2D convolution with 2D filter in order to excavate deep features at first. We expect to find more local features of the data. However, it has been discovered that the 2D feature is not obvious in the network traffic data. Figure 5 shows the performance of 1D convolution and 2D convolution acting on network traffic, respectively.

According to our understanding of network traffic data, our experimental data consist of a series of hexadecimal numbers, which is expounded in Section 4.1. From Figure 5c,d, it can be concluded that 1D filters only consider the relationship between the front and back bits of hexadecimal data of each sample, but 2D filters take the feature aggregation of a square region into account. In terms of professional experience, there are no obvious interactions between the areas of traffic data and the business feature data composed of different kinds of features. Theoretically, 1D convolution will have a better effect on malicious behavior detection task with PCAP files and business feature data. Of course, the experiment results also prove this idea in Section 4.5.

## 4. Experiments and Results

### 4.1. Data Description

In this paper, we used four datasets to verify the proposed detection fusion methods. They are NB15 [39], CIC2012 [40], CIC2017 [41] and VPN2016 [42]. There are two kinds of original data in each dataset, namely PCAP files and business feature data. PCAP files are the basic form to store network communication behaviors and contain almost all the original information of a network communication. Business feature data files are collected by business feature collection system for which its features are designed by experts. From the perspective of bearing information, PCAP files utilize more original information in the process of network connection. However, more information content also represents that it is more likely to contain useless messages and have a bad influence on model training. Business feature data pay more attention to the most effective data characteristics that are more precise, explicit and interpretable. In terms of content, they can complement each other. Then, we will introduce the structure of two kinds of data and depict each dataset in detail.

#### 4.1.1. PCAP Files

**Figure 6:**
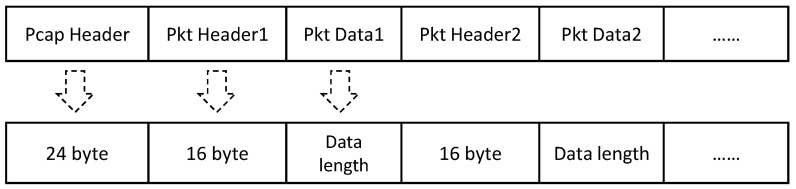
The structure of a PCAP file which consists of a PCAP header, a number of data packets with the packet header and packet content.

PCAP files can be translated into a group of hexadecimal numbers. Data segments at specific locations represent different meanings that may explain connection source, connection destination, data length and other connection information. Figure 6 depicts the structure of a PCAP file. Generally, the length of PCAP header of a PCAP file is 24-byte, which contains byte order information, file version number, timestamp accuracy and so on. The content of the packet header is relatively fixed. It always describes the timestamp with an 8-byte, the length of data frame with an 8-byte, namely Caplen, and the length of offline data with an 8-byte. Then, actual transmission data follow the length Caplen defined by packet header.

#### 4.1.2. Business Feature Data

It has been reported that business feature data contain many useful business features. Table 2 describes some types of feature. Flow features record the overall situation of a network connection or session, which mainly includes some of five-tuple information. Base features contain some basic information in people’s cognition, including the duration of a network connection, the number of dropped packets and etc. Content features display some information about transmitting data. Time features portray the important content related to time. Generated features are calculated to achieve a specific purpose, for example, some of them are sorted accordingly with the last time feature to capture similar characteristics of the connection records for each 100 connections that are sequentially ordered.

#### 4.1.3. Datasets Overview

NB15 [39] is generated by the Australian Centre for Cyber Security (ACCS) with PerfectStorm. It contains nine types of network attacks and one type of normal network behavior data. Each category contains a different number of samples from 100 to 20,000, which constitute an unbalanced dataset. In addition, the ratio of training and test data amount is 7:3.

VPN2016 [42] is exposed by University of New Brunswick (UNB). They captured a session over VPN, and the categories are described as follows. TraP2P labels identify file-sharing protocols such as Bittorrent. Chat labels identify instant-messaging applications. VoIP labels represent the Voice over IP label that groups all traffic generated by voice applications. Streaming labels identify multimedia applications that require a continuous and steady stream of data. Email labels are generated using a Thunderbird client and Alice and Bob Gmail accounts. File-transfer labels identify traffic applications for which its main purpose is to send or receive files and documents.

CIC2012 [40] and CIC2017 [41] simulated different types of cyber attacks which are generated by UNB. CIC2017 contains fourteen categories of attack network traffic and one category of normal traffic. CIC2012 contains five attack categories in CIC2017. We attempt to combine CIC2012 with CIC2017 in order to make up a new dataset. Considering that the experiment results have already achieved a perfect performance, we plan to make a new dataset to test the identification ability of fusion models on the data with a certain distribution difference. Finally, this new dataset comprises 15 kinds of data type, including a normal type and other attack categories. There are about 10,000 samples in each category, and the category that numbers the least comprises about 2000 samples. Tt can be concluded from Section 4.3.1 that fusion models also make a good performance on the new one.

### 4.2. Data Processing

**Figure 7:**
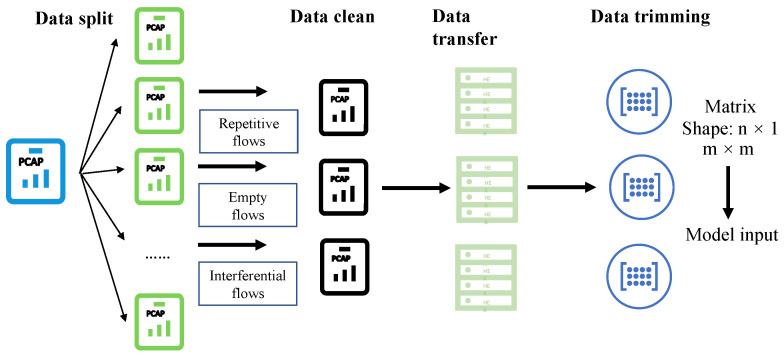
Data processing steps for PCAP files with data split, data clean, data transfer and data trimming.

In this paper, we need to deal with two kinds of raw data, PCAP files and business feature data. From the perspective of processing steps, it is more complicated to handle PCAP files than business feature data. As for business feature data, we need to convert characters to numerical variables and normalize the numerical value for each feature. Then, the processed data can feed into the model. We used Figure 7 to explain the process of data processing.

As Figure 7 shows, four steps before the data enters the detection model are data split, data clean, data transfer and data trimming. Firstly, we split PCAP files into flows based on five-tuple, including a source IP address, a source IP port, a destination IP address, a destination IP port and the protocol in use. Then, we filter out some invalid information, namely repetitive flows, empty and interferential flows by data cleaning process. Another step is data transfer that extracts hexadecimal code from PCAP files. Finally, we trim the data to an appropriate size for one dimension CNN or two dimension CNN inputs.

Due to the different length of data, it is impossible to send all data into the model for training. In the experiment, we work out that the average number of packets per flow divided by five-tuple is about two in datasets. Thus, we chose the average length of 784 bytes as the processing length of each stream. If it is longer than 784, it will be intercepted. If it is shorter than 784, it will be filled with zero. The header, transmission control protocol information and payload are concerned here except for IP information. IP information is only used as data split, which ensures that IP information that may have label properties will not be brought into the training data. When processing the PCAP file, we extracted the data of the corresponding location and sent it into the model. In this process, we only care about the data distribution. It does not affect the whole process of data processing whether the data are encrypted or not. As for the division of training set and test data set, we randomly divided the data into a training set and test set according to the ratio of 7:3 on dataset VPN2016 and CIC. Dataset NB15 has been divided into training and test data on the official website and can be used directly. The number of training set and test set of each dataset is depicted in Table 3. After the data processing steps, we can train our models and verify the results.

### 4.3. Results

In this section, we will compare the effects among the proposed detection fusion models and previous papers’ experiment results. In addition to the proposed fusion model results, we discuss the different detection results between one-dimension-based CNN structure and two-dimension-based one. We calculate the five evaluation indexes to evaluate the effects of the models: Macro-f1, Weighted-f1, Recall, Precision and Accuracy. We use $O_{prec}$, $O_{recall}$, $O_{acc}$, $O_{macro-f1}$ and $O_{weighted-f1}$ to denote them. The calculation processed can be described as follows:

(9) $$O_{prec}=\frac{1}{N}\sum_{i=1}^{N}\frac{TP}{TP+FP},$$

(10) $$O_{recall}=\frac{1}{N}\sum_{i=1}^{N}\frac{TP}{TP+FN},$$

(11) $$O_{acc}=\sum_{i=1}^{N}\frac{TP}{TP+FP},$$

(12) $$O_{macro-f1}=\frac{1}{N}\sum_{i=1}^{N}\frac{2\times O_{prec}\times O_{recall}}{O_{prec}+O_{recall}},$$

(13) $$O_{weighted-f1}=\sum_{i=1}^{N}w_{i}\frac{2\times O_{prec}\times O_{recall}}{O_{prec}+O_{recall}},$$

where *N* is the number of categories in the dataset, *TP* denotes the number of correctly identified positive samples, *TF* denotes the number of correctly identified negative samples, *FP* refers to the number of wrongly identified positive samples, *FN* refers to the wrongly identified negative samples and $w_{i}$ represents the weight of this category to the total data quantity.

**Figure 8:**
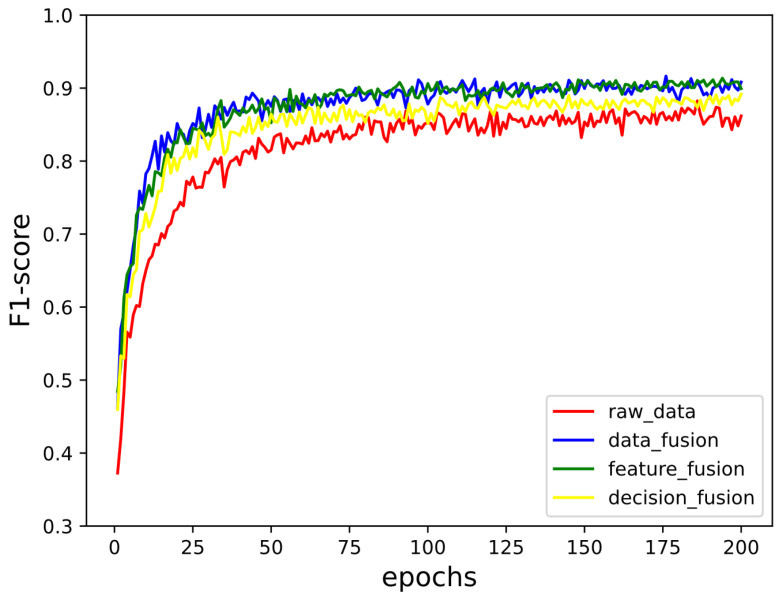
The changes of F1-score in the training process on VPN2016.

In the experiment, we use the Adam optimizer to optimize the training results. The learning rate is set to 0.001 in order to make our models perform the best, and the batch size is 128 here. We trained about 200 epochs to obtain the best results. Figure 8 shows the changes of F1-score in the training process on VPN2016. The training processes of other datasets are similar to this one. It can be concluded that the improvements of the result become slow after 50 epochs and nearly do not change after 100 epochs.

#### 4.3.1. Overall Performance on Datasets

In this paper, we select four different datasets to compare the performances of each model in order to verify the advancement and rationality. We chose two kinds of baseline results on each dataset. Some recent previous methods training on these datasets are collected, and the simple model without any fusion structure is considered in Table 4, Table 5 and Table 6.

Table 4 shows the experiment results of the proposed models and comparative models on dataset NB15. We compared six previous methods based on DL methods on NB15. It can be summarized from this table that the feature fusion model attains the highest score in each index. Compared with the worse previous models, fusion models can reach over 10% improvement. Furthermore, in contrast to our optimized simple model, fusion models also make some significant improvements. According to the analysis of the results, all fusion methods are effective with a growth rate of 2 to 5%. In addition, we compared some traditional ML-based methods, Logistic Regression (LR), KNN, DT, RF and eXtreme Gradient Boosting (XGBoost). The accuracy of these methods is much lower than the proposed fusion ones.

Table 5 shows the experiment results on dataset VPN2016. The table also shows that fusion models made some improvements. Unlike NB15, the best performance comes from the decision fusion method. Table 6 shows detection results on the mixed dataset with CIC2012 and CIC2017, and the results show that feature fusion has the best performance. It can be concluded that even if the accuracy of a single model is very high, the fusion model can still improve the effect and achieve better results. At the same time, the comparative traditional method results are also depicted in Table 5 and Table 6, which are far less than the results of the fusion ones.

As Tables show, some previous methods have achieved good performance. However, they are not comprehensive enough from the perspective of data understanding. Yang et al. [43] used business features extracted by expert experience to complete the classification tasks. Janarthanan and Zargari [46] proposed a feature selection framework on traffic network. Kim and Chung [49] and Zhou et al. [53] also paid more attention on relatively important features, which may ignore some information that has an impact on the results. This is also the advantage of our proposed fusion models. In addition, some DL-based models [44,45,52] are too simple to achieve good performance. Moreover, some 2D convolution-based CNN [47,48], RNN-based [50] and LSTM-based [51] models may be a little complex for the analysis of network traffic data and can reduce generalization ability. In this paper, the proposed 1D-convolution-based fusion model can balance these problems and achieve ideal results. Different from images, network traffic data and business feature data do not have obvious regional features. Thus, 2D-convolution-based kernels may dig out some features that do not have relevance and have a bad impact on the model. Beyond that, fusion models can combine different information provided by at least two data formats and have stronger recognition ability. We also discuss their advantages in detail in Section 4.4 and Section 4.5.

#### 4.3.2. Details of Each Fusion Method Results

**Figure 9:**
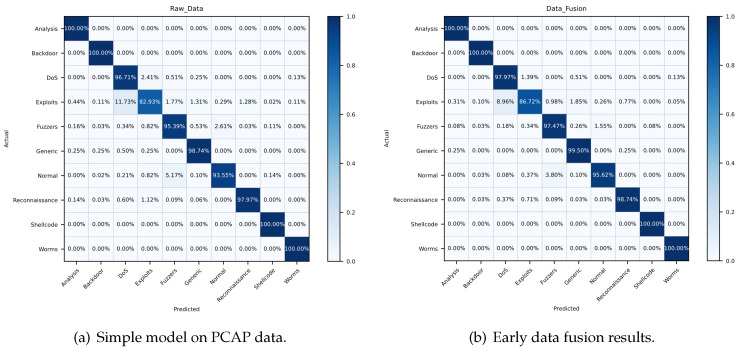

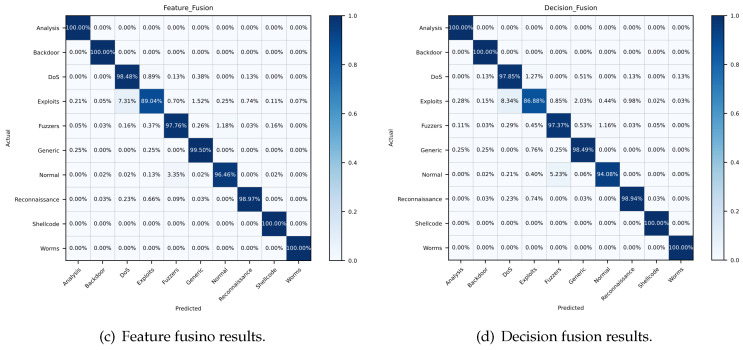
Confusion matrices of detection results on dataset NB15. True labels are on vertical axis and predicted labels are on horizontal axis.

**Figure 10:**
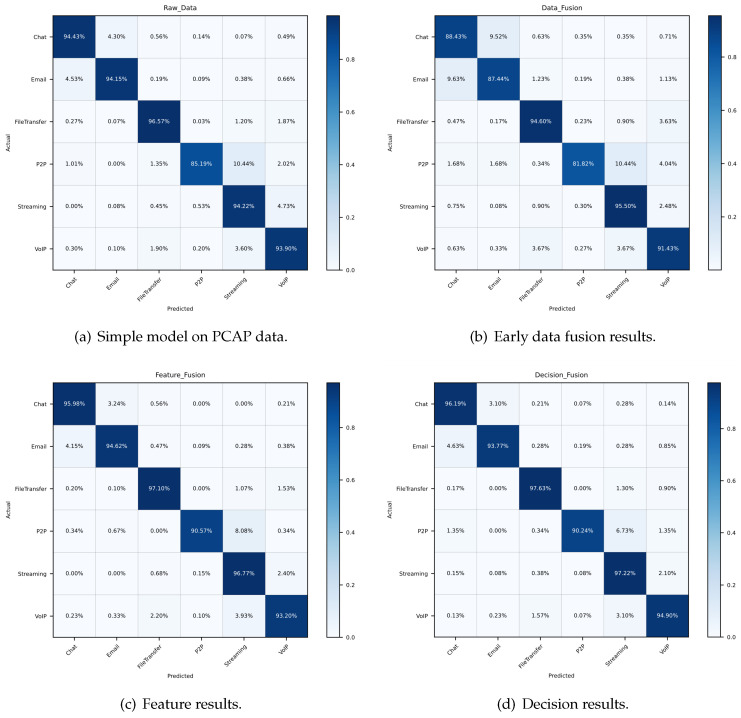
Confusion matrices of detection results on dataset VPN2016.

**Figure 11:**
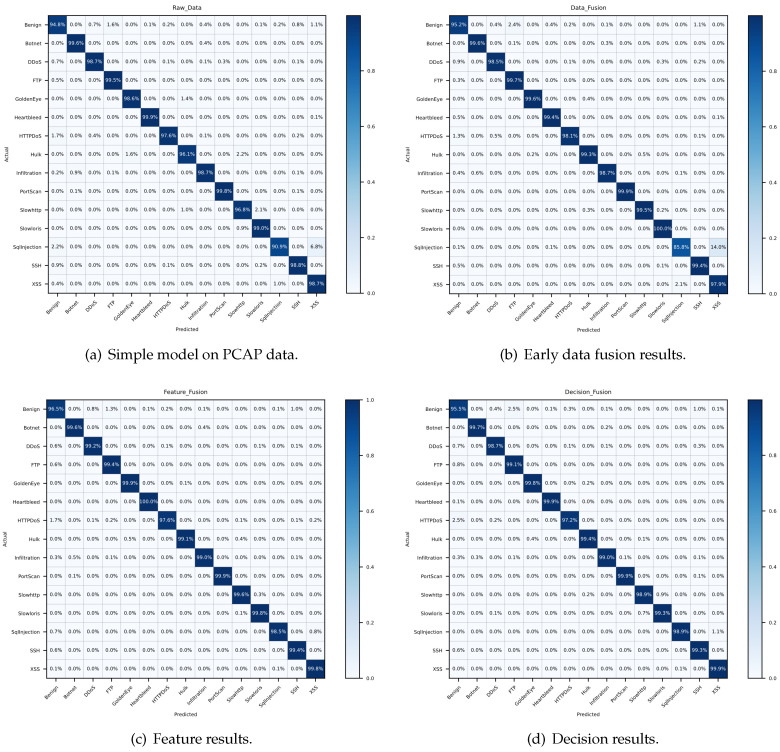
Confusion matrices of detection results on dataset CIC.

In order to depict the results in some more details, we calculate the evaluation indexes of each category. Figure 9, Figure 10 and Figure 11 reveal the confusion matrices of simple model, early data fusion model, feature fusion model and decision fusion model on dataset NB15, VPN2016, CIC2012 and CIC2017. In Figure 9, there are ten categories with a kind of normal behavior and nine attack behaviors. From the comparison of Figure 9a and the best fusion model in Figure 9c, the accuracy rates of all categories in feature fusion results are improved or flat. The maximum increase in a single category reached 6%. This claimed that the feature fusion model has a positive impact on every subclass. Early data fusion and decision fusion also make sense here, but the effect is weaker than the feature fusion model. Figure 10 portrays six different types results of VPN network behavior. As shown in the figure, two fusion models worked except for the early data fusion one. Moreover, the decision fusion model has the best performance on this data set. Figure 11 displays 15 categories with a benign subclass and other malicious types. Figure 11c describes that the accuracy of all categories made some progress, except for FTP behavior.

**Figure 12:**
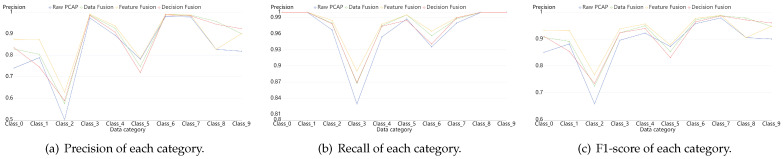
Classification results of each category on dataset NB15. They are composed of Precision, Recall and F1-score, which are calculated by a simple model with PCAP data, data fusion model, feature fusion model and decision fusion model.

**Figure 13:**
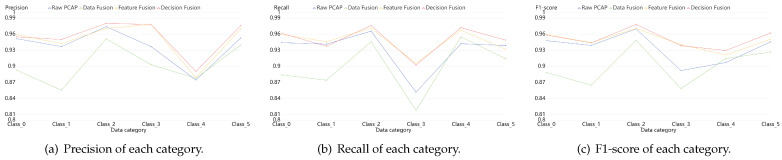
Classification results of each category on dataset VPN2016. They are composed of Precision, Recall and F1-score which are calculated by a simple model with PCAP data, data fusion model, feature fusion model and decision fusion model.

**Figure 14:**
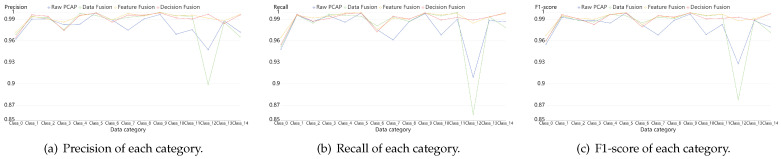
Classification results of each category on dataset CIC. They are composed of Precision, Recall and F1-score which are calculated by a simple model with PCAP data, data fusion model, feature fusion model and decision fusion model.

For the sake of clarity, we depicted Figure 12, Figure 13 and Figure 14. They compared the effects among simple model and fusion mudels more obviously. They portrayed the results of three datasets: NB15, VPN2016 and the mixed one, CIC. From the figures, we can draw a conclusion that fusion models are better than the simple one on the identification results of each sub category, especially the performance of Feature fusion and Decision fusion models denoted with yellow lines and red lines in the figures.

### 4.4. Discussion of Fusion Models

From Section 4.3.1 and Section 4.3.2, we introduce the experiment results of the proposed methods. From the performance result point of view, the feature fusion model is the best one that achieves the best result on two datasets, and the decision fusion model achieves the best performance on another dataset. Consequently, we can conclude that, compared with the early fusion method, the late and deep fusion process can obtain a better result.

The early data fusion process occurs at the beginning of the detection process, which combines the raw data directly without any further processing. It can be regarded as adding human business experience to the raw data. The input information increases and provides more dimension features for the training process. From the point of experiments, in the wake of the increasing content of raw data, model has the opportunity to mine more data distribution characteristics. The results show that early data fusion improved on dataset NB15, CIC2012 and CIC2017 but performed worse on dataset VPN2016. Early data fusion is the shallowest fusion process, which means that the composite data only directly stitches the original data of different distributions. It may affect the original good data separability and reduce the cognitive ability of the model. Consequently, it shows better discrimination against some datasets but worse on others. In this case, the feature fusion methods and decision fusion methods are considered here.

The feature fusion process arises after feature generation in the malicious behavior detection model. In this paper, we attempt this process before the last fully connected layer. Before feature-level fusion, both raw datasets go through the same CNN-based network structure. By this means, two different kinds of raw data are more likely to be transformed into a similar feature vector space. Then, stitched data with feature fusion can express the data content preferably. With regard to the decision fusion process, it is not involved in data registration and looks similar to a more independent fusion process. The results show that decision fusion always performs better than data fusion and sometimes performs better than feature fusion. On the whole, later fusion methods are obviously better than the early one, which illustrates that the data trained by models have a more similar data distribution. These later fusion methods can obtain more deeper features and weaken the difference between the original input data.

Above all, fusion models usually play a significant role in promoting detection results. According to different scenes and data, we can choose the most suitable fusion method. We should consider not only accuracy but also efficiency in practical application. Therefore, we also evaluated models’ efficiencies. As for the testing process of malicious behavior detection, each method consumes several milliseconds, which fully meet the efficiency of practical application. In the training process, the original simple model takes 58.86 s to complete a training round with 70,000 pieces of sample. Under the same experimental setting, the training consumptions of data fusion, feature fusion and decision fusion are 62.03 s, 65.55 s and 65.89 s, respectively. In the case of the same resource usage, there is little difference in their computational efficiency. Therefore, we do not have to worry too much about efficiency in the model fusion.

### 4.5. One-Dimensional and Two-Dimensional Convolution-Based Malicious Behavior Detection Results Comparation

**Figure 15:**
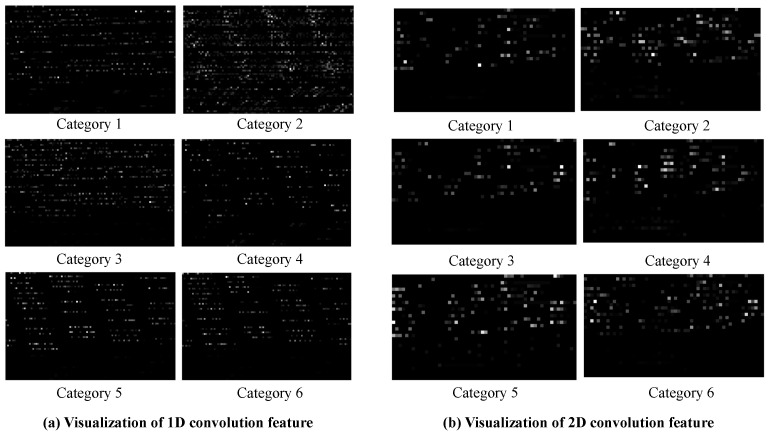
Visualization of 1D and 2D dimensional convolution feature maps.

In order to illustrate the rationality of convolution structure and to further verify the difference between 1D convolution and 2D convolution in current malicious behavior detection task, we compared some feature outputs during the training process. Figure 15 shows the visualization of 1D and 2D dimensional convolution feature maps of six different categories in dataset VPN2016. We plan to compare the feature grayscale images output from convolutional layers in the model. Figure 15a describes 1D results, and (b) describes the 2D results. Compared with the images, network traffic has limited association information in each point. Thus, we used the 1D filter and extracted more data characteristics in (a). Figure 15b paid attention to square area features and captured less information that contains fewer pixels here.

**Figure 16:**
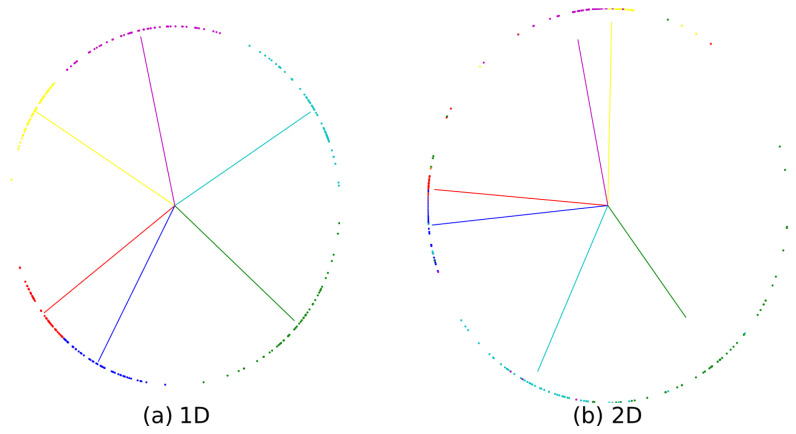
Experiment results on dataset VPN2016 used 1D convolution and 2D convolution. The interval of each point represents the interval between samples.

We simultaneously tested feature extraction results of the two kinds of convolutions in the training process. Figure 16 depicts the sample distribution converted into a two-dimensional plane on hyperspheres. The points on the circle denote testing samples, and each color refers to a different category. It is obvious that Figure 16a has a smaller intraclass compactness and larger interclass discrepancy after 1D convolution model training, which is consistent with our theoretical inference. Quite evidently, models will be more discriminating in this situation.

## 5. Conclusions

In this paper, we proposed three different kinds of fusion models based on one-dimensional convolution neural networks for malicious network behavior detection. At the input of each fusion framework, we used packet capture (PCAP) files and business feature data. Then, we extracted distinct and complementary features from the input modalities by applying 1D-CNN-based models and fused these features at more than one stage in our three novel fusion frameworks. The results output from experiments on several datasets proved that the proposed fusion methods are effective on malicious network behavior detection tasks. From the experimental results, feature fusion and decision fusion models have an outstanding performance in terms of detection accuracy, which can improve the results over 5% in the case of almost no increase in computing time. We consider these fusion frameworks to be an important step forward on malicious network behavior detection when there are different kinds of original data for us to utilize.

In the future, we will continue to design some effective methods for malicious network behavior detection and verify their application values in the actual application network environment. Although the proposed models are simple and lightweight, there is still room for improvement to meet real-time requirements. Moreover, we will further study the interpretability of the models in malicious behavior detection tasks, and the visualization of feature maps is a basic work. We will attempt to build a description system of network traffic in order to identify network behavior more intuitively and accurately.

## Figures and Tables

**Table 1:** Some related methods in the past three years.

Ref.	Method	Description	Type
[15] 2019	RF	Method that solves data imbalance.	ML
[16] 2019	DT, RF, KNN	An adaptive voting algorithm.	ML
[17] 2021	DiFF-RF	Random partitioning binary trees.	ML
[18] 2019	Cluster, RF	Process highly imbalanced data.	ML
[19] 2017	Genetic algorithm	Improve accuracy and efficiency.	ML
[20] 2019	SVM	Improve accuracy.	ML
[21] 2020	CNN	Outstanding performance.	DL
[22] 2020	CNN, GAN	Data augmentation, imbalanced data.	DL
[23] 2021	CNN, image-based	Improve accuracy, interpretable model.	DL
[24] 2018	Text-CNN, RF	Good classification results.	DL
[25] 2019	VAE, fingerprinting	Good identification representations.	DL
[26] 2020	AE, end-to-end	Imbalanced classification.	DL
[27] 2021	AE, CNN	Few-shot malicious traffic detection.	DL
[28] 2021	CNN, LSTM	Feature representations, accuracy.	DL
[29] 2020	Genetic, DNN	Improve accuracy, interpretable.	DL
[30] 2019	VAE, DNN	Outstanding classification performance.	DL
[31] 2020	Transformer	Improve efficiency and accuracy.	DL
[32] 2021	Transformer	The dynamic word embedding used.	DL
[33] 2020	GAN, LSTM	Data augmentation, improve accuracy.	DL

**Table 2:** Features list in business feature data.

Feature Type	Feature Contents
Flow features	Source port number, Destination port number, Protocol, …
Base features	Record total duration, Source to destination time to live, …
Content features	Source TCP window advertisement, Source TCP sequence number, …
Time features	Record start time, Source inter-packet arrival time, …
Generated features	Some statistical or calculated value

**Table 3:** Samples number of training set and test set.

Dataset Name	Type	Number
Dataset NB15	Training set	175,341
	Test set	82,332
Dataset VPN2016	Training set	125,744
	Test set	53,890
Dataset CIC2012, CIC2017	Training set	634,229
	Test set	271,812

**Table 4:** Experiment results and comparation of data NB15.

Type	Method	Prec	Recall	Acc	F1-Macro	F1-Weight
Previous Methods	DualNet, Yang et al. [43]	-	-	0.833	-	-
	Khan et al. [44]	-	-	0.8913	-	-
	Vinayakumar et al. [45]	0.623	0.66	0.66	0.596	
	Yang et al. [30]	0.7743	0.9739	0.8597	0.8627	
	Janarthanan et al. [46]	-	-	0.8162	-	-
	Khammassi et al. [19]	-	-	0.8142	-	-
Comparative Methods	LR	0.3089	0.3483	0.4225	0.3419	0.4697
	KNN	0.5582	0.5593	0.5602	0.5586	0.5593
	DT	0.7896	0.7756	0.7880	0.7680	0.7902
	RF	0.7775	0.7762	0.7766	0.7768	0.7766
	XGBoost	0.6749	0.6781	0.6762	0.6764	0.6759
Proposed Methods	Simple (PCAP)	0.8278	0.9653	0.9183	0.8822	0.9228
	Simple (Feature)	0.6130	0.8116	0.7969	0.6593	0.8169
	Early data fusion	0.8697	0.9760	0.9410	0.9132	0.9440
	Feature fusion	**0.8790**	**0.9802**	**0.9514**	**0.9221**	**0.9534**
	Decision fusion	0.8616	0.9736	0.9369	0.9073	0.9397

**Table 5:** Experiment results and comparation of data VPN2016.

Type	Method	Prec	Recall	Acc	F1-Macro	F1-Weight
Previous Methods	Guo et al. [47]	-	-	0.9292	-	-
	Song et al. [48]	0.876	0.873	-	0.875	
Comparative Methods	LR	0.2684	0.2025	0.2305	0.2280	0.2399
	KNN	0.6864	0.6719	0.6800	0.6715	0.6812
	DT	0.8458	0.8404	0.8419	0.8416	0.8488
	RF	0.8526	0.8521	0.8552	0.8491	0.8557
	XGBoost	0.8000	0.8493	0.8134	0.8046	0.8132
Proposed Methods	Simple (PCAP)	0.9379	0.9307	0.9458	0.9338	0.9459
	Simple (Feature)	0.6011	0.5842	0.5843	0.5766	0.5702
	Early data fusion	0.9036	0.8987	0.9179	0.9005	0.9180
	Feature fusion	0.9500	0.9471	0.9529	0.9479	0.9531
	Decision fusion	**0.9551**	**0.9499**	**0.9594**	**0.9519**	**0.9596**

**Table 6:** Experiment results and comparation of data CIC2012 and CIC2017.

Type	Method	Prec	Recall	Acc	F1-Macro	F1-Weight
Previous Methods	Kim et al. [49]	0.94	0.94	-	0.94	
	Le et al. [50]	0.9475	0.975	-	0.9708	
	Sun et al. [51]	-	-	0.9844	-	-
	Ferrag et al. [52]	-	-	0.9823	-	-
	Zhou et al. [53]	-	-	0.968	-	-
	Vinayakumar et al. [45]	0.972	0.962	0.962	0.965	
Comparative Methods	LR	0.1113	0.0275	0.0418	0.0378	0.0410
	KNN	0.5995	0.6031	0.5970	0.5969	0.5970
	DT	0.9504	0.9498	0.9499	0.9496	0.9504
	RF	0.9393	0.9398	0.9411	0.9393	0.9401
	XGBoost	0.9065	0.9111	0.9012	0.9039	0.9067
Proposed Methods	Simple (PCAP)	0.9809	0.9783	0.9823	0.9795	0.9823
	Simple (Feature)	0.7072	0.6344	0.6288	0.6470	0.6495
	Early data fusion	0.9817	0.9803	0.9871	0.9814	0.9871
	Feature fusion	**0.9924**	**0.9915**	**0.9926**	**0.9919**	**0.9926**
	Decision fusion	0.9908	0.9897	0.9906	0.9902	0.9906

## Data Availability

The datasets used in this paper are available online [39,40,41,42], and they are also available from the corresponding author upon request.
